# Can a visual self-learning tool improve immunisation awareness? Evidence from a quasi-experimental pilot study in Madhya Pradesh, India

**DOI:** 10.1186/s12889-025-25318-z

**Published:** 2025-11-19

**Authors:** Priyanka Das, Santosh Shukla, Mehak Bhatia, Nitin Kothari, Harkabir Singh Jandu, Divya Monga, Mahesh Kumar Aggarwal

**Affiliations:** 1 National Health Mission, Government of Madhya Pradesh, Bhopal, India; 2 William J. Clinton Foundation, New Delhi, India

**Keywords:** Immunization, Health education, Community health workers, Health knowledge, Attitudes, Practice, Program evaluation, Pilot projects

## Abstract

**Introduction:**

Immunisation coverage in low- and middle-income countries continues to face challenges due to persistent gaps in vaccination schedule knowledge among caregivers and health workers. The Routine Immunisation Wheel was developed as a low-cost visual tool to complement existing systems like Mother Child Protection (MCP) cards and community healthcare worker visits in Madhya Pradesh, India. This pilot study evaluates the tool’s effectiveness on improving knowledge and awareness among caregivers and frontline workers (FLWs), as well as its potential for scaling within routine immunisation programs.

**Methodology:**

Using a mixed-methods approach, we conducted a quasi-experimental pilot comparing intervention and control districts with 384 caregivers per arm and more than 60 FLWs (vaccinators/Auxiliary Nurse Midwife (ANMs), mobilizers Accredited Social Health Activists (ASHAs) and Anganwadi Workers (AWWs). Statistical analysis included descriptive statistics for demographics, mean scores with 95% confidence intervals for knowledge domains, independent samples t-tests for group comparisons, chi-square tests for proportions, and thematic analysis of qualitative feedback.

**Results:**

At endline, caregiver knowledge scores in the intervention district were higher than at baseline for immunisation schedule (mean: 53.7% vs. 35.6%, *p* < 0.05), immunisation benefits (mean: 87.4% vs. 62.5%, *p* < 0.05), and session site awareness (95.1% vs. 89.8%, *p* < 0.05). When compared with the control district at endline, the intervention district had, on average, higher scores for immunisation schedule knowledge, immunisation benefits and session site awareness (all *p* < 0.05). The tool demonstrated high utility among health workers, with 100% adoption by ANMs for due listing and counselling.

**Conclusion:**

The routine immunisation wheel effectively addresses critical knowledge gaps in routine immunisation through its dual functionality as both caregiver reminder and healthcare worker job aid. These results highlight its potential for integration into broader immunisation strategies, especially in low-literacy, resource-limited settings.

**Supplementary Information:**

The online version contains supplementary material available at 10.1186/s12889-025-25318-z.

## Background

The Universal Immunisation Programme (UIP), launched in 1985, has played a pivotal role in reducing India’s morbidity and mortality due to vaccine-preventable diseases (VPDs), representing one of the world’s largest public health initiatives [1]. While the country has made remarkable progress in immunisation coverage over recent decades, with full immunisation coverage (FIC) for children aged 12–23 months improving from 62% (National Family Health Survey (NFHS)−4) to 76.4% (NFHS-5, 2019-21), significant subnational inequities persist, leaving vulnerable populations behind [2–4]. These gaps are most evident among zero-dose (ZD) children, with India reporting the second-highest absolute number of ZD children worldwide in 2023 [5, 6]. Similarly, measles vaccination rates highlight areas for improvement, with India ranking among the top 10 countries with a high number of children missing this critical vaccine [5]. However, national averages mask considerable heterogeneity across states, districts, and socioeconomic groups. In Madhya Pradesh (MP), for instance, 22.79% children aged 12–23 months do not complete their full basic vaccination schedule (missing any of the eight vaccine doses recommended in the first year of life), while 12% of this age group do not receive the Measles Rubella 1 st dose (MR1) vaccine [6]. Compounding this challenge, districts with higher under-vaccination prevalence tend to show greater within-district heterogeneity, indicating more pronounced inequities in vaccine access and uptake at the local level.

Barriers to immunisation in India operate at multiple levels. While individual-level factors like maternal education and socioeconomic status are well documented, equally critical are health system challenges - particularly frontline health workers’ (FLWs’) capacity constraints [7]. Many Accredited Social Health Activist (ASHAs) and Auxiliary Nurse Midwife (ANMs), despite their crucial role in community mobilisation and vaccine administration, lack required communication skills to effectively address caregiver concerns or counter vaccine hesitancy [8]. This gap becomes particularly apparent when handling vaccine-related rumours or questions about side effects, as evidenced in Haryana where workers reported feeling unprepared for such conversations [9]. These systemic barriers in health worker capacity compound the individual-level challenges, creating complex obstacles to achieving universal immunisation coverage.

Recognising these multifaceted challenges, India has implemented various strategies to improve immunisation awareness and uptake, employing diverse communication channels including mass media, community meetings, radio, and local theatre. Evidence suggests that the most effective interventions employ multiple complementary approaches that foster dialogue and directly address knowledge gaps among both health workers and caregivers [10–13]. Such comprehensive strategies have demonstrated success in improving vaccination attitudes, increasing uptake, enhancing coverage, and reducing dropout rates across various settings [10–13].

**Fig. 1:**
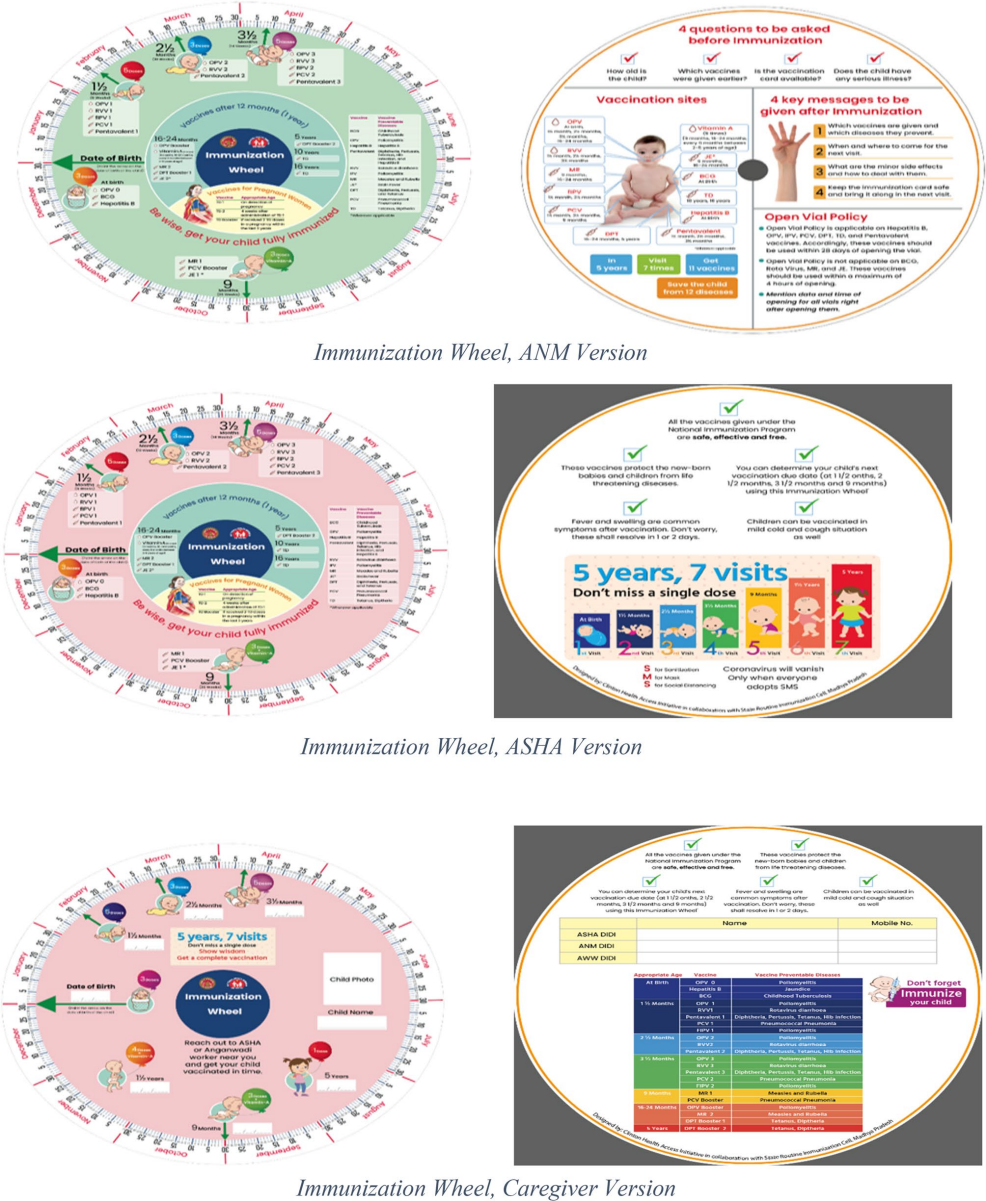
Routine Immunisation Wheel

It is within this context that the Government of Madhya Pradesh, in collaboration with the William J. Clinton Foundation (WJCF), developed the innovative Routine Immunisation (RI) Wheel (*Tikakaran Chakra* in Hindi; “Chakra” refers to the inner wheel of the Routine Immunisation Wheel tool, with “Chakra” being the Hindi term for “wheel”) - a tailored solution designed to address the specific immunisation challenges faced by the State. This self-learning tool serves dual purposes: for caregivers; it functions as an intuitive reminder system for vaccination schedules, while for health workers it enhances counselling capabilities and builds technical capacity. The routine immunisation wheel features an interactive rotating disc that visually maps vaccination due dates to the National Immunisation Schedule, while displaying essential information including vaccine-specific details (administration routes, preventable diseases), key counselling messages for pre-vaccination screening, and post-vaccination care instructions (Fig. 1).

### Components and features of Routine Immunisation (RI) wheel

The Routine Immunization (RI) Wheel, or *Tikakaran Chakra*, is a visual, rotatable calendar tool designed to help frontline health workers (FLWs) such as ANMs, ASHAs, as well as caregivers, ensure timely and complete vaccination of children. By aligning the wheel’s central arrow with a child’s date of birth, users can instantly view due dates for the five vaccination touchpoints in the first year of life, with subsequent doses displayed in the centre panel. The wheel also lists vaccine names, the diseases they prevent, administration routes, vaccination sites on the child’s body, and key pre- and post-vaccination messages, making it both a scheduling aid and a comprehensive IEC (Information, Education and Communication) tool. Its bright, relatable (uniform-matched) colors and clear layout support use in low-literacy settings and address common challenges such as identifying due vaccines, closing gaps in message delivery, and limited availability of IEC/IPC (Interpersonal Communication) materials.

The RI Wheel is available in three versions tailored to different user groups. The ANM version supports due-list preparation and accurate counselling during RI sessions, with prompts for screening, key messages and follow-up. The ASHA (Mobilizer) version is suitable for household visits and Village Health Sanitation and Nutrition Days (VHSNDs), helping mobilizers explain schedules and encourage vaccine uptake. The caregiver version, distributed at delivery points or during ASHA’s home visits, enables families to independently track their child’s vaccination schedule and understand the benefits of timely immunization.

In practice, ANMs use the RI Wheel during outreach RI sessions and at health facilities to quickly determine and communicate the next due date and deliver the key immunization messages and benefits. ASHAs carry the wheel during mobilization to demonstrate due dates, address hesitancy, and remind families about vaccination’s importance. During interactions with caregivers, FLWs communicated the next session date whenever the scheduled due date and the actual session date differed. For subsequent doses, particularly in cases of delayed vaccine administration, FLWs advised caregivers to align the arrow on the RI Wheel with the actual date of vaccination rather than the birth date, enabling more accurate calculation of the next due date. Caregivers keep their version at home to check future due dates and mark them on calendars or set reminders.

Taken together, the RI Wheel is intended to streamline due-list preparation and counselling for FLWs (ANMs, ASHAs, and Anganwadi Workers (AWWs) and to provide caregivers with a simple visual reminder of upcoming doses. This paper describes the pilot implementation in MP and presents a pilot evaluation comparing immunisation-related knowledge among caregivers and FLWs in a district where the wheel was introduced versus a control district.

## Study objectives

The objectives of study were.

- To assess the tool’s impact on improving caregivers’ knowledge and awareness of immunisation’s importance and schedule.
- To assess the tool’s impact on enhancing FLWs’ (ANMs, ASHAs, and AWWs) knowledge about vaccinations and their ability to communicate immunisation messages effectively.

## Methodology

### Study design

A quasi-experimental design with intervention and control districts.

### Study setting

The study was conducted in two districts of Madhya Pradesh, India.

### Sample size & sampling approach

It involved purposive sampling approach based on selected indicators. We selected two comparable and representative districts in Madhya Pradesh based on three criteria:

- Full Immunisation Coverage (FIC) [14]
- Proportion of the tribal population to ensure demographic representation.
- Literacy rates, as anecdotal evidence and design testing indicated that literacy levels significantly influence the uptake of interventions [15]

### District selection

Harda district (population: 570,465) was selected as the intervention site due to the presence of an existing programmatic network through WJCF, which facilitated permissions, logistics, and training. To enable meaningful comparison, a district with similar FIC, female literacy, and tribal population percentages was sought. Based on these parameters, Hoshangabad, Katni, and Ratlam emerged as the most comparable to Harda. Considering operational feasibility, Hoshangabad district (population: 1,241,350) was chosen as the control district due to its proximity and similarity in key demographic and programmatic indicators.

### Block selection

Each district contains 3–7 administrative blocks. Within each district, two blocks were selected using tribal population percentage and female literacy rate as the primary similarity criteria, ensuring alignment with state averages. In Hoshangabad, Seoni-Malwa (tribal population 24%, female literacy 54%) and Kesala (40%, 57%) closely matched Harda’s Harda block (27%, 51%) and Khirkiya block (37%, 47%). Timarni block in Harda (37%, 50%) was excluded to prevent potential intervention spillover, as it shares a border with Hoshangabad.

### Final selection

- Intervention blocks (Harda district, 3 blocks total): Harda (population: 157,272) and Khirkiya (149,476).
- Control blocks (Hoshangabad district, 7 blocks total): Seoni-Malwa (168,101) and Kesala (129,551).

**Fig. 2:**
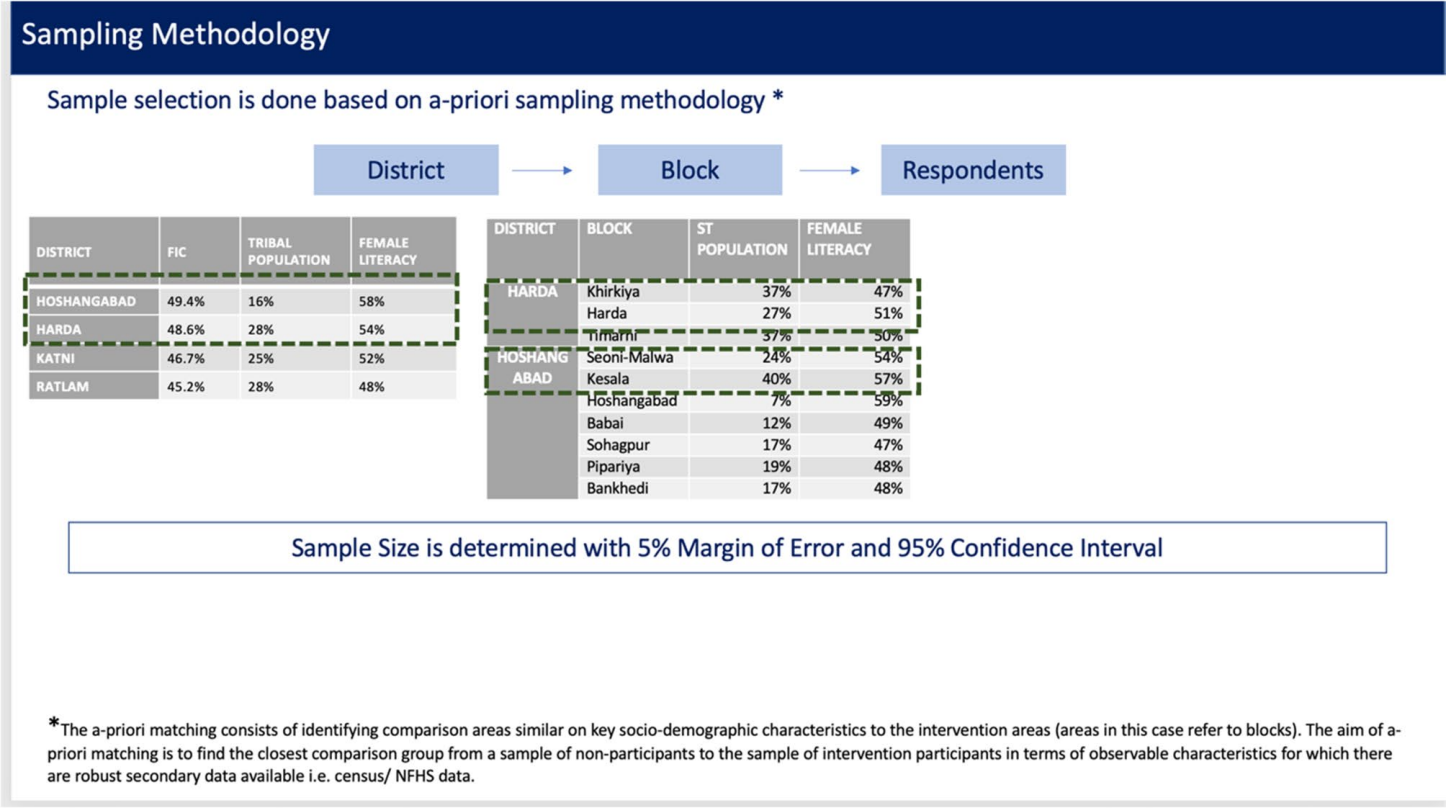
Sample Selection Methodology

Blocks were selected to be geographically distant within their respective districts to minimise contamination effects. (Fig. 2)

Sample Size Calculation: For both intervention and control arms, sample size was calculated using the formula below:

$$n=(z^2\times p\times(1-p))/e^2$$

Where

- n = Required sample size
- Z = Z-value corresponding to the desired confidence level (1.96 for a 95% confidence level)
- p = Estimated prevalence (proportion) of knowledge regarding vaccination benefits and schedule (0.5 for 50% prevalence)
- e = Margin of error (0.05 for ± 5% margin of error)

Using this formula, the required sample size was determined to be 384 caregivers per study district. Data was collected from an equal number of caregivers in each study arm. This sample of 384 caregivers was distributed across two blocks in each district. Within each block, the sample of 192 caregivers was further divided across 10 randomly selected Sub Health Centres (SHCs), the most peripheral health facilities in the public system, serving as the first point of contact for basic preventive and primary healthcare in rural areas, with an upper limit of 20 caregivers per SHC to ensure the sample was evenly spread and not concentrated in larger SHCs. Villages were randomly selected by field investigators from the list provided by the ANM. Field Investigators obtained the target cohort list (primary caregivers with children aged 0–9 months) from ASHAs or AWWs, who maintained this information in the Reproductive and Child Health (RCH)/Immunisation register, and randomly selected respondents from this list. The inclusion criteria for caregivers to be respondents were primary caregivers with children aged 0–9 months.

All ANMs of the selected SHCs and, ASHAs, and AWWs of the selected villages were included in the study, as their total numbers in the target areas were limited (e.g., typically 1 ANM per SHC, 1–2 ASHAs/AWWs per village). This census approach ensured complete representation of FLWs involved in immunisation delivery across intervention and control sites.

The same sampling framework was applied for both baseline and endline assessments. However, different caregiver respondents were evaluated in the baseline and endline, as children who were 0–9 months old during the baseline had aged by the time the endline was conducted. Furthermore, there was variability in the number of respondents interviewed in the baseline and endline. The field investigators were given the target of interviewing a sample size plus 20% extra respondents to account for the dropping of respondents during the data cleaning phase.

#### Data collection

Baseline and endline surveys were conducted using close-ended questionnaires. (Supplementary File I) Data was collected from caregivers of children aged 0–9 months, as well as FLWs including ANMs, ASHAs, and AWWs, to assess their knowledge of immunisation benefits, schedule, location, and vaccine safety. Survey tools were tailored to each respondent category:

- Caregivers: 48-item questionnaire
- Mobilizers (ASHAs/AWWs): 21-item questionnaire
- Vaccinators (ANMs): 21-item questionnaire

Questions were grouped into thematic domains to evaluate knowledge systematically.

In addition to knowledge outcomes, the endline survey also captured information on the actual use of the RI Wheel by caregivers, mobilisers, and vaccinators. Respondents were asked whether they had received and used the wheel, and, if so, how they applied it in practice (e.g., calculating due dates, counselling, preparing due lists).

All tools were pre-tested and translated into the local language. Data collection was carried out on tablets using pre-coded questions, with Google Forms serving as the online platform for data collection. To accommodate areas with low or no internet connectivity, field investigators first recorded responses on physical copies of the forms and later entered the data into the digital platform when network access was available. The tools used were an original assessment instrument designed by the authors for this study. As it was a newly developed, non-proprietary tool, no copyright license was required for its use. The timeline for data collection and intervention implementation is outlined below:

- Baseline Evaluation: February 2021
- Intervention Period: March–July 2021
- Endline Evaluation: August–September 2021

The details of respondents from whom data was collected is provided in Table 1.

**Table 1:** Study sample size for pilot study

District	Respondents	Baseline	Exposure	Endline
Intervention	Caregivers	413	~ 9k	427
	Vaccinators	10	55	12
	Mobilisers	52	350	83
Control	Caregivers	389	×	412
	Vaccinators	18	×	15
	Mobilisers	44	×	48

*ANMs* Auxiliary Nurse Midwives (vaccinators), *ASHAs* Accredited Social Health Activists, *AWWs* Anganwadi Workers (ASHAs and AWWs collectively referred to as mobilizers)

#### Data quality measures

To ensure high data quality, all field investigators received structured training on the study objectives, questionnaire content, and standardised interviewing techniques. Field supervisors conducted spot checks and directly observed a sample of interviews daily to ensure adherence to protocols. Completed questionnaires were reviewed at the end of each day for completeness and internal consistency. Automated range and logic checks were applied during data cleaning to identify outliers or inconsistencies, which were resolved through review of source data and clarification with field teams where needed.

#### Tool Roll-Out

The tool was implemented through a structured training and distribution process:

- Training: WJCF staff and government master trainers conducted initial training sessions for FLWs (AWWs, ASHAs, ANMs) on tool usage. Quarterly review meetings included re-orientation to reinforce learning.
- Tool Distribution:
  - Vaccinator and mobilizer versions were provided during training sessions.
  - Caregiver versions were distributed via birth delivery centres and ASHAs during community outreach.

This ensured sustained engagement and proper utilisation across all levels.

#### Data analysis

Data were analysed using IBM SPSS Statistics (Version 25.0; IBM Corp., Armonk, NY, USA), originally developed as the Statistical Package for the Social Sciences. Demographic characteristics of respondents were analysed using descriptive statistics. For caregivers, the study tool covered four knowledge domains:

1. Immunisation Schedule: Questions covering the timing and age-appropriate doses of key childhood vaccines (e.g., BCG) and the total number of recommended vaccination visits. (9 items)
2. Immunisation Benefits: Items assessing awareness of the role of vaccines in preventing specific diseases and improving child health. (2 items)
3. PW (Pregnant Women) Vaccines: Questions on recommended antenatal vaccines, including tetanus-diphtheria (TD). (2 items)
4. Vaccine Safety: Items covering knowledge of common post-vaccination reactions, safety practices, and the importance of completing vaccination despite mild side effects. (3 items)

For vaccinators and mobilizers, two domains were assessed: Immunisation Schedule (5 items) and General immunisation program (definition, key messages, number of childhood vaccines, and vaccine-preventable diseases; 6 items in the vaccinator tool and 4 in the mobiliser tool).

All items were dichotomously scored (1 = correct, 0 = incorrect). For questions not inherently right/wrong (e.g., *“Can you tell me what are these key messages?”*), responses were scored as “1” if the participant was able to recall all required key messages accurately and “0” otherwise. Responses such as “don’t know” and non-responses (missing values) were also coded as “0” to maintain consistency in scoring. This approach ensured that domain scores reflected only demonstrated knowledge.

Thus, for each domain, we summed correct responses and expressed performance as a mean percentage score (sum/number of items) × 100, with 95% confidence intervals (CI).

In addition to the knowledge domains, the tool also included items on caregiver awareness, specifically knowledge of immunisation session sites and health worker (HCW) contact information. These items were dichotomously coded (1 = aware, 0 = not aware) and analysed as binary outcomes using chi-square tests to assess pre–post differences within groups and between-group comparisons at endline.

For continuous outcomes (domain percentage scores), independent-samples t-tests compared pre–post groups within districts and intervention vs. control at endline; between-group contrasts are reported as mean differences with standard errors (SE) and p-values. To enhance comparability, we also report within-group pre–post changes alongside between-group endline differences.

For the usability of the RI Wheel at endline, usage patterns among caregivers, mobilisers, and vaccinators were analysed descriptively to complement the knowledge outcome analysis.

#### Ethical considerations

Ethical approval for the present study was obtained from Sigma Institutional Review board (IRB) under reference number: 10,005/IRB/21–22, dated 10 May 2021. Written informed consent was obtained from all participants prior to their inclusion in the study. Verbal consent was obtained from participants who were illiterate. All participants were informed of their right to withdraw at any time. Participants were assured of anonymity and voluntary participation.

## Results

### Socio-demographic characteristics of caregivers

The survey findings indicated that a majority of caregivers across baseline and endline assessments in both intervention and control group, were between 22 and 29 years of age. Furthermore, 78% to 84% of the surveyed caregivers reported having 1–2 children at the time of data collection. The age distribution of their children was approximately 25% had infants aged 1–3 months, 36% had children aged 3–6 months, and 29% had children aged 6–9 months. The sample comprised three frontline worker categories: ANMs (16–29%), ASHAs (37–48%), and AWWs (34–39%), with proportions remaining stable between pre- and post-intervention periods across both study arms (Table 2).

**Table 2:** Socio-demographic characteristics of respondents in Pilot study

		Intervention		Control	
Caregivers		Pre (%) (*n* = 413)	Post (%) (*n* = 427)	Pre (%) (*n* = 389)	Post (%) (*n* = 412)
Caregiver’s Age	18–21	17	12	20	19
	22–25	52	51	47	44
	26–29	26	27	27	24
	30–34	4	9	5	11
	> 35	1	1	1	2
Caregivers’ No of children	1	42	33	43	46
	2	42	49	35	33
	3	11	14	16	15
	4 or 4+	5	4	6	6
Age of the child (in months)	0–1	9	8	7	8
	1–3	20	26	32	28
	3–6	38	40	32	34
	6–9	32	25	29	30
	> 9	1	1	0	0

		**Pre (%)** (*n* = 62)	**Post (%)** (*n* = 95)	**Pre (%)** (*n* = 62)	**Post (%)** (*n* = 63)
Vaccinators and Mobilisers	ANM (Vaccinators)	16	13	29	24
	ASHA (Mobilisers)	48	48	37	41
	AWW (Mobilisers)	35	39	34	35

Vaccinators = ANMs who administer vaccines, Mobilizers = ASHAs/AWWs who support vaccine awareness

### Knowledge of caregivers

A significant improvement was observed in the intervention district across domains, such as knowledge of the immunisation schedule and immunisation benefits. Knowledge of the immunisation schedule increased from 35.56% (95% CI- 33.6–37.5) at baseline to 53.7% (95% CI- 51.9–55.4) post-intervention (*p* < 0.05), while knowledge of immunisation benefits rose from 62.5% (95% CI- 58.9–66.2) to 87.4% (95% CI- 85.1–89.8) (*p* < 0.05). No significant change was observed for knowledge regarding Tetanus Diptheria (TD) vaccine given to pregnant woman (PW), which increased slightly from 53.2% (95% CI: 51.2–55.3) to 55.2% (95% CI: 53.2–57.2) (*p* = 0.253). For vaccine safety, the intervention district showed no meaningful change, with scores moving from 61.6% (95% CI: 59.2–64.03) to 61.3% (95% CI: 58.2–64.3) (*p* = 0.765).

In the control district, smaller but statistically significant gains were observed for the immunisation schedule (28.1%, 95% CI: 26.5–29.6 to 33.3%, 95% CI: 31.7–35.0; *p* < 0.05))and vaccine safety (67.7%, 95% CI: 66.1–69.3 to 71.2%, 95% CI: 68.7–73.3; *p* = 0.02), while no significant changes occurred for immunisation benefits or PW vaccines.

Post intervention, the intervention had a significant positive effect on improving knowledge of the immunisation schedule (+ 20.3%, SE = 1.2, *p* < 0.05) and immunisation benefits (+ 35.5%, SE = 2.0, *p* < 0.05). No significant effect was found for PW vaccine knowledge (+ 2.2%, SE = 1.4, *p* = 0.126). For vaccine safety, the intervention district showed no change (61.6% to 61.3%, *p* = 0.765); however, because the control district improved significantly, the endline comparison indicated a relative negative effect for the intervention (–9.9%, SE = 1.9, *p* < 0.05).

Overall, the intervention was effective in improving knowledge of the immunisation schedule and benefits, but had no impact on PW vaccine knowledge and was associated with a relative decline in vaccine safety perceptions compared to the control district. This conclusion is based on the endline comparison between intervention and control districts (Table 3).

**Table 3:** Mean scores (%) on knowledge related to immunisation among caregivers

	Mean Scores % (CI)							
	Intervention District			Control District			Endline Intervention- Control Difference	
Knowledge Regarding/Time	Pre (*n* = 413)	Post (*n* = 427)	Δ (*p* value)	Pre (*n* = 389)	Post (*n* = 412)	Δ (*p* value)	Mean Difference (SE)	*p* value
Immunisation Schedule	35.5 (33.6–37.5)	53.7 (51.9–55.4)	18.2 (< 0.05)	28.1 (26.5–29.6)	33.3 (31.7–35.0)	5.2 (< 0.05)	20.3 (1.2)	< 0.05
Immunisation Benefits	62.5 (58.9–66.2)	87.4 (85.1–89.8)	24.9 (< 0.05)	50.2 (47.7–52.7)	51.9 (48.7–55.1)	1.7 (0.419)	35.5 (2.0)	< 0.05
PW vaccines	53.2 (51.2–55.3)	55.2 (53.2–57.2)	2.0 (0.253)	53.2 (51.7–54.6)	53.0 (50.9–55.1)	−0.02 (0.892)	2.2 (1.4)	0.126
Vaccine Safety	61.6 (59.2–64.03.2.03)	61.3 (58.2–64.3)	−0.3 (0.765)	67.7 (66.1 − 69.3)	71.2 (68.7–73.3)	3.5 (0.02)	−9.9 (1.9)	< 0.05

*CI* Confidence Interval, *SE* Standard Error, Δ = Pre–Post Difference (Post – Pre)

### Awareness among caregivers

The study assessed changes in caregiver awareness regarding immunisation session sites and healthcare worker (HCW) contact information across intervention and control districts. In the intervention district, awareness of session sites significantly increased from 79.4% pre-intervention to 95.1% post-intervention (*p* < 0.05), while the control district showed a smaller increase from 82.5% to 89.8% (*p* = 0.03). Post intervention, the intervention showed significant change in intervention district as compared to the control (*p* < 0.05). Similarly, awareness of how to contact an HCW improved significantly in the intervention district from 84.7% to 97.7% (*p* < 0.05), whereas in the control district, it declined from 92.0% to 85.9% (*p* = 0.007), with a significant change in intervention district as compared to the control (*p* < 0.05). These findings indicated that the intervention was effective in enhancing caregiver awareness of immunisation services, particularly in improving knowledge of session sites and HCW contact mechanisms. (Table 4)

**Table 4:** Proportion of caregivers on session site awareness and HCW contact information

	% of caregivers						
	Intervention District			Control District			Endline Intervention- Control
Awareness Regarding/Time	Pre (*n* = 413)	Post (*n* = 427)	*p* value	Pre (*n* = 389)	Post (*n* = 412)	*p* value	*p* value
Session site	79.4	95.1	< 0.05	82.5	89.8	0.03	0.004
Contacting HCW	84.7	97.7	< 0.05	92.0	85.9	0.007	< 0.05

#### Knowledge among mobilisers

The knowledge assessment among mobilisers included two key domains: knowledge regarding immunisation schedule and knowledge regarding general immunisation programme. For the knowledge regarding immunisation schedule, the intervention district showed a marked increase from 84.6% (95% CI: 79.2–89.9) at baseline to 98.1% (95% CI: 96.7–99.2) post-intervention (*p* < 0.05). In the control district, scores also improved from 68.8% (95% CI: 63.7–72.6) to 77.1% (95% CI: 70.7–83.4) (*p* = 0.025). Post-intervention, the independent samples t-test confirmed that the intervention district had significantly higher knowledge scores than the control district (mean difference = 21.1%, SE = 2.5, *p* < 0.05).

In the knowledge regarding general immunisation program, the intervention district’s score rose slightly from 76.15% (95% CI: 71.47–80.8) to 79.2% (95% CI: 77.8–80.7), though this change was not statistically significant (*p* = 0.131). In the control district, scores declined from 83.6% (95% CI: 78.6–88.5) to 77.9% (95% CI: 73.2–82.5) (*p* = 0.094). The post-intervention difference between districts was not significant (mean difference = 1.3%, SE = 2.0, *p* = 0.498).

Overall, total knowledge scores in the intervention district improved significantly from 79.9% (95% CI: 75.6–84.2) to 87.6% (95% CI: 86.5–88.8) (*p* < 0.05), while the control district showed no significant change (76.7% to 77.5%, *p* = 0.767). Post-intervention comparison confirmed that mobilisers in the intervention district scored significantly higher overall than those in the control district (mean difference = 10.1%, SE = 1.6, *p* < 0.05). (Table 5)

**Table 5:** Mean scores (%) with confidence interval (CI) on knowledge related to immunisation among mobilisers

	Mean Scores % (CI)							
	Intervention District			Control District			Endline Intervention- Control Difference	
Knowledge Regarding/Time	Pre (*n* = 52)	Post (*n* = 83)	Δ *p* value	Pre (*n* = 44)	Post (*n* = 48)	Δ *p* value	Mean Difference (SE)	*p* value
Immunisation Schedule	84.6 (79.2–89.9)	98.1 (96.7–99.2)	13.5 (< 0.05)	68.8 (63.7–72.6)	77.1 (70.7–83.4)	8.3 0.025	21.1 (2.5)	< 0.05
General Immunisation program	76.15 (71.47–80.8)	79.2 (77.8–80.7)	3.1 (0.131)	83.6 (78.6–88.5)	77.9 (73.2–82.5)	−5.7 (0.094)	1.3 (2.0)	0.498
Total Scores	79.9 (75.6–84.2)	87.6 (86.5–88.8)	7.7 (< 0.05)	76.7 (73.4–80.1)	77.5 (73.5–81.5)	0.8 (0.767)	10.1 (1.6)	< 0.05

*CI* Confidence Interval, *SE * Standard Error, Δ = Pre–Post Difference (Post – Pre)

#### Knowledge among vaccinators

In the intervention district, vaccinators showed a significant improvement in general immunisation programme knowledge, increasing from 70.3% (95% CI: 54.9–85.7) at baseline to 98.6% (95% CI: 95.5–101.6) post-intervention (*p* < 0.05). Total scores also improved significantly, rising from 76.7% (95% CI: 66.2–87.3) to 96.2% (95% CI: 91–101.4) (*p* = 0.01). Knowledge regarding the immunisation schedule increased from 84.4% (95% CI: 74.2–94.6) to 93.3% (95% CI: 85.1–101.6), but this change was not statistically significant (*p* = 0.142).

In the control district, no statistically significant changes were observed in any of the domains between pre and post-intervention. Post-intervention comparisons between districts showed that vaccinators in the intervention district scored significantly higher than those in the control district for general immunisation programme knowledge (mean difference = 33.1%, SE = 8.5, *p* = 0.001) and total scores (mean difference = 19.2%, SE = 6.02, *p* = 0.004), while no significant difference was found for immunisation schedule knowledge (mean difference = 2.6%, SE = 4.9, *p* = 0.598). (Table 6)

**Table 6:** Mean scores (%) with confidence interval (CI) on knowledge related to immunisation among vaccinators

	Median Scores % (IQR)							
	Intervention District			Control District			Endline Intervention- Control Difference	
Knowledge Regarding/Time	Pre (*n* = 10)	Post (*n* = 12)	Δ (*p* value)	Pre (*n* = 18)	Post (*n* = 15)	Δ (*p* value)	Mean Difference (SE)	*p* value
Immunisation Schedule	84.4 (74.2–94.6)	93.3 (85.1- 101.6)	8.9 (0.142)	91.1 (84.1–98.1)	90.6 (83.5–97.7)	−0.5 (0.926)	2.6 (4.9)	0.598
General vaccination program	70.3 (54.9–85.7)	98.6 (95.5–101.6.5.6)	28.3 (< 0.05)	51.8 (43.8–59.8)	65.5 (49.3–81.7)	13.7 (0.097)	33.1 (8.5)	0.001
Total Scores	76.7 (66.2–87.3)	96.2 (91–101.4.4)	19.5 (0.01)	69.6 (64.1–75.2)	76.9 (66.2 - 87.7)	7.3 (0.188)	19.2 (6.02)	0.004

*CI* Confidence Interval, *SE* Standard Error, Δ = Pre–Post Difference (Post – Pre)

#### Use of the immunisation wheel

At endline, the usability of the RI Wheel was also assessed.

##### Caregivers

Approximately 85% of caregivers correctly calculated vaccination due dates in the intervention district. Around 68% showed the RI Wheel to field investigators during the endline survey. Among them, 93% received the wheel from mobilisers (ASHA or AWW), 5% from institutional delivery points, and 2% from ANMs. Of those who used the wheel, 85% could determine the next vaccination due date, while only 10% were unaware of its use.

##### Mobilisers

52% of mobilisers incorporated the wheel into counselling. The majority (39%) used it for determining due dates, 31% for counselling on routine immunisation, 19% for creating due lists, and 6% for spreading government’s Information, Education, and Communication (IEC) messages.

##### Vaccinators

All ANMs used the immunisation wheel, with 100% providing due dates and 92% sharing immunisation-related information (vaccines, due dates, VPDs, AEFI). Additionally, 75% used it to prepare due lists.

## Discussion

This mixed-methods evaluation of Madhya Pradesh’s Routine Immunisation Wheel intervention revealed important insights about improving vaccination knowledge in low-resource settings. The study addresses a critical gap in India’s immunisation system, where, despite high awareness of vaccine benefits [16, 17], caregivers often lack practical knowledge about vaccination schedules, a finding consistent with research across low- & middle- income countries (LMICs) [18, 19]. The Routine Immunisation wheel successfully bridged this gap by providing a tangible, visual aid that complemented existing reminder systems like Mother Child Protection (MCP) cards and ASHA visits.

The pilot study phase demonstrated significant improvements in caregivers’ knowledge about immunisation schedules and benefits in intervention areas as compared to control. These findings align with successful implementations of similar tools globally, such as Thailand’s wheel calendar and Pakistan’s tracker bracelets, while addressing their limitations through context-specific design [13, 20]. Broader communication interventions show a similar pattern of domain-specific effects such as Kilkari’s immunisation content increased men’s (but not women’s) knowledge, and tailored mobile voice messages such as mMitra improved maternal vaccine knowledge [17, 21]. Home-visit counselling has likewise raised awareness of tetanus causes, symptoms, and prevention, even when beliefs about vaccine efficacy remained unchanged [22]. Evidence from the systematic review by Dudeja et al. indicates that technology-based interventions, ranging from mobile reminders to m-health apps and interactive voice systems, are most effective when paired with strong FLW engagement [23]. In line with this, the cluster randomised trial by Ruchit Nagar et al. in rural Udaipur found that a digital pendant and voice reminder platform, when integrated with FLW support, significantly improved infant immunisation adherence [24]. Our results reinforce this principle by showing that even without digital technology, a low-cost, context-specific tool like the RI Wheel, when embedded within ASHA/ANM-led counselling during VHSNDs and home visits, can deliver comparable gains in targeted knowledge domains. The RI Wheel’s design provides a constant, tangible point of reference, overcoming literacy barriers, particularly important in MP, where 27% of women are illiterate (NFHS-5) and sustaining engagement without the need for technological infrastructure [4]. 

Our findings align with broader evidence that enhancing caregiver awareness and strengthening FLW capacity can translate into improved immunisation outcomes. Studies from South Asia have shown that targeted maternal education and communication strategies can increase both coverage and timeliness of childhood vaccination [12, 22]. Interventions such as redesigned immunisation cards coupled with centre-based education have reduced dropout rates and improved completion of vaccine schedules [13]. Large-scale mobilization efforts, such as India’s Social Mobilization Network originally developed for polio, further demonstrate how FLW capacity-building can strengthen routine immunisation uptake [10]. 

Our pattern, gains in immunisation schedule and benefits knowledge, but not PW vaccines and vaccine safety, mirrors this literature’s domain-specific effects. By contrast, the absence of a significant change in PW vaccination knowledge in our study may be due to the RI Wheel’s primary use at the point of delivery and post-delivery, rather than during pregnancy, which limited reinforcement during antenatal care. An ANC-focused version and integration with pregnancy registers may be needed to address this gap. Similarly, the unexpected decline in vaccine safety knowledge among caregivers may reflect the unique context of the COVID-19 pandemic, during which attention was heavily focused on COVID-19 vaccination drives, potentially overshadowing routine immunisation safety communication. Furthermore, the 85% correct usage rate among caregivers suggests that the wheel’s visual format is both acceptable and practical in real-world settings.

The intervention RI Wheel did not show a measurable effect on vaccinators’ knowledge of the immunisation schedule compared to the control group. This may be partly due to the small vaccinator sample size, which limited the statistical power to detect smaller effects, and partly due to the COVID-19 period, when vaccinators’ primary focus and workload were directed towards pandemic-related vaccination activities. It is also possible that vaccinators already had strong baseline knowledge of the schedule, so the added value of the tool for them may lie more in improving counselling related to return dates and enhancing efficiency in rescheduling, outcomes that were beyond the scope of this study.

The intervention rollout provided valuable implementation lessons. Unlike digital tools like Kilkari, an Interactive Voice Response System (IVRS) delivering maternal-child health messages via automated calls, which have demonstrated variable effectiveness over time [17], the RI Wheel’s physical format offered sustained engagement. Similar to Khushi Baby, an organisation leading a programme using wearable digital necklaces to track child immunisation, the RI wheel’s success relied on seamless integration with FLWs’ routines. This aligns with Scott et al. findings, who underscores community health workers’ role as critical health information intermediaries [25]. 

The study’s findings also reflect several key implications of the COVID-19 pandemic. The difference observed in vaccine safety knowledge did not represent a true decline in the intervention district, instead, it reflects an improvement in the control district that was not replicated in intervention areas, possibly due to other concurrent activities that were not captured in this study. These unexpected gains in the control district may also relate to heightened vaccine awareness generated by pandemic response efforts, though this requires further investigation [26, 27]. Importantly, the pandemic highlighted the RI wheel’s reliability as a non-digital solution during health system disruptions, with 68% of caregivers still retaining and using it. 

Two key strengths distinguish this intervention from similar health communication tools. First, its visual, less-text based design overcomes literacy barriers that often limit the effectiveness of traditional health education materials. Second, its dual functionality - serving as both a caregiver reminder system and health worker job aid - creates multiplicative effects within the health system. The RI Wheel’s resilience during pandemic disruptions, maintaining 68% caregiver retention despite health system challenges, further demonstrates its robustness as a low-tech solution in resource-limited settings.

The study also revealed important implementation challenges that inform future scale-up efforts. While ANMs achieved near-universal adoption (100%), mobilisers showed more variable engagement (52%), likely due to their broader responsibilities and increase in pandemic-related workload. This differential uptake suggests the need for role-specific adaptations in future iterations. These findings also underscore the importance of accounting for external factors and ensuring robust monitoring during future rollouts.

Taken together, these findings highlight the RI Wheel’s distinct advantages over other reminder systems: combining tactile engagement with visual simplicity at minimal cost, and integrating effectively into existing immunisation programmes. While feasibility and scale-up will be discussed in a separate article, these evaluation results provide important lessons for future applications, including potential adaptation for other scheduled health interventions such as antenatal care or tuberculosis treatment.

### Limitations

However, several limitations must be acknowledged when interpreting these findings. First, the six-month evaluation period, while sufficient to measure knowledge outcomes, was too brief to assess long-term behavioural change or vaccination coverage impacts, a constraint noted in similar mobile health interventions like Kilkari that showed variable effects over time. Future research should assess whether knowledge gains observed in our study translate into improved immunisation coverage and timeliness, as has been documented in other caregiver-awareness and FLW capacity-building interventions. Second, the survey tool used in this study was newly developed and, although pre-tested for clarity and comprehension, it had not undergone a formal validation process. Some questions may have been directive and certain constructs potentially biased, which could limit reliability. Future studies should incorporate a systematic validation process to enhance robustness. Third, in our scoring approach, all non-responses (“missing values”) were coded as incorrect (‘0’) to ensure consistency and maintain comparability across domains. While this method prevents inflation of knowledge scores, it may slightly underestimate true knowledge levels if non-response was due to factors other than lack of knowledge. Fourth, the quasi-experimental design, though pragmatic for real-world evaluation, limits causal attribution. In particular, the intervention district (Harda) also hosted the earlier design-testing activities. Although different blocks were used for design testing and the pilot, and pilot sampling explicitly excluded design-testing sites, spillover/priming across blocks cannot be ruled out, and any interaction with existing programmatic support in Harda could bias estimates upward. To avoid over-interpretation, we present results as associations and avoid causal claims. Fifth, the findings’ generalizability may be limited to similar rural, low-literacy settings, but the intervention may need adaptation for different settings. The absence of literacy data for caregivers prevents analysis of how education levels may have mediated tool effectiveness, despite qualitative evidence suggesting good usability across literacy strata. Additionally, data on participant characteristics such as wealth, education, and ethnicity were not collected, which limits the ability to examine how these factors may have influenced knowledge outcomes or differed between intervention and control groups. Sixth, the limited sample size for vaccinators, combined with the COVID-19 period when their primary focus was on pandemic-related vaccination efforts, reduced the statistical power to detect meaningful improvements in vaccination schedule knowledge within this group, underscoring the need for larger studies specifically examining health workers’ outcomes. Seventh, the sampling frame was based on targeted cohort lists prepared by ASHAs and AWWs. While operationally feasible and consistent with existing programme systems, this approach may have limited the inclusion of truly missed communities and zero-dose children, those most likely to have the lowest knowledge and awareness levels, thereby potentially underestimating the intervention’s relevance for these high-priority groups. Finally, we did not systematically track all concurrent activities in either district, unmeasured co-interventions may have influenced outcomes.

### Future Research Directions and Policy Implications

Future research should explore several critical areas, including longitudinal studies to evaluate the Routine Immunisation Wheel’s impact on vaccination timeliness and coverage and link caregiver or health worker knowledge measures to administrative immunisation records (e.g., RCH/eVIN) to test whether gains in knowledge translate into improved uptake and timeliness. Additional areas include cost-effectiveness analysis comparing it to other reminder systems, and assessments of how socioeconomic factors influence its uptake and use. While the RI wheel has been scaled statewide, its performance and alignment with the country’s digital health initiatives during scale-up have not been formally assessed; future research should address this gap. The RI Wheel, as a ready reckoner, provides all essential pre- and post-vaccination messaging prescribed in national guidelines for RI sessions. While certain features of the tool can be incorporated into emerging digital health platforms such as UWIN, it remains unlikely that digital systems alone can fully substitute the face-to-face counselling functions of frontline health workers. Nevertheless, digital integration offers opportunities to complement the tool, for example, the state government is considering a digital RI Wheel on its website to gamify due-date tracking and engage citizens more broadly. As countries focus on restoring and strengthening immunisation services in the post-pandemic era, the routine immunisation wheel presents a promising, scalable solution. By leveraging context-specific health communication, it has the potential to empower both caregivers and health workers, thereby reinforcing routine immunisation systems.

## Conclusion

The Routine Immunisation Wheel improved caregiver and health worker knowledge of vaccination schedules and benefits in Madhya Pradesh, demonstrating its value as a low-cost, scalable tool for LMICs. Its successful statewide rollout to 80,000 frontline workers highlights practical advantages, including adaptability and integration with existing systems. While short-term knowledge gains were significant, further research should assess long-term impacts on vaccination coverage and timeliness. The study provides a replicable model for scaling evidence-based health interventions in resource-limited settings. Policymakers should consider adopting this tool to strengthen routine immunisation programs, particularly in low-literacy areas. The wheel exemplifies how simple, context-appropriate solutions can bridge critical gaps in healthcare delivery.

## Supplementary Information

Supplementary Material 1.

## Data Availability

The datasets generated and analyzed during the current study are not publicly available due to organisational policies and ethical considerations. Data may be available from the corresponding author upon reasonable request and with written permission from government counterparts.
