# Supplementary material for: Can a visual self-learning tool improve immunisation awareness? Evidence from a quasi-experimental pilot study in Madhya Pradesh, India
Source: BMC Public Health. 2025 Nov 19;25:4036. doi: 10.1186/s12889-025-25318-z (PMC12628981; doi:10.1186/s12889-025-25318-z)
Supplement: Supplementary file 1 — Supplementary Material 1. [file 12889_2025_25318_MOESM1_ESM.pdf]

# Can a Visual Self-Learning Tool Improve Immunization Awareness? Evidence from a Quasi-Experimental Pilot Study in Madhya Pradesh

## Supplementary File I

## PILOT STUDY TOOLS

# ANM RI Wheel Survey

1. Name of District:-
   a. Harda
   b. Hosangabad

2. Block Name

   a. Kharikiya
   b. Hardah/Handiya
   c. Shivani-Malwa
   d. Keshla

3. Village Name…………………

4. ANM Name………………

5. Name of Surveyor…………………

6. Consent given

   a. Yes
   b. No

7. Can you tell me till what age children are vaccinated?
   a. Correct (5 or 16 years)
   b. Wrong
   c. Don't know
   d. can not tell

8. Can you tell me what is Vaccination? (Vaccination is the process by which a person is made immune or resistant against an infection, usually by administering a vaccine, which subsequently induces the person's own immune system to protect them against the disease.)
   a. Yes
   b. No
   c. May be

9. Are any questions asked to the mother before administering the vaccine?
   a. Yes
   b. No
   c. May be
   d. Can not tell

10. What questions are asked to the mother before applying the vaccine?
    a. Age of child
    b. What were the previous Vaccine given to the children
    c. Vaccination card is available
    d. Does the child have any disease
    e. Don't Ask
11. Can you tell me when children are given vaccinations in the first year?(Mark all the time stated)
    a. month
    b. 2.5 month
    c. 3.5 month
    d. 9 month
    e. Don't know

12. Can you tell me when the child is given vaccinations after the first year? (Mark all the time stated)
    a. 16-24 Months
    b. 5 years
    c. 10 years
    d. 16 years
    e. Don't know

13. Can you tell me how many times a mother has to come to get her child vaccinated in 5 years(5 saal 7 baar)
    a. Correct ( 6 ya 7)
    b. Wrong
    c. Don't know

14. Do you know when to give TD booster dose to a pregnant woman?
    a. Correct (If you become pregnant again within 3 years after taking TD in previous pregnancy)
    b. Wrong
    c. Don't know

15. Can you tell me how many vaccines are given to a child in his lifetime? Including drop vaccine.
    a. 11
    b. Not 11
    c. Don't know

16. Can you tell how many diseases a child can be protected from by all these vaccines?
    a. 12
    b. Not 12
    c. Don't Know

17. Do you know of any important message that should be given to a beneficiary?
    a. Yes
    b. Don't Know

18. How many such messages should you given?
    a. 4
    b. Not 4
    c. Don't know

19. Can you tell me what are these key messages ?
    a. Which vaccine was taken today and which disease will it protect you from?
    b. Next date and place of vaccination
    c. Common side effects and their treatment
    d. Keep the vaccination card safe and bring it with you the next time you come
    e. Don't know

20. Is there any policy regarding Vaccine Vials?
    a. Yes
    b. No
    c. Don't Know

21. Can you tell me what the policy says?
    a. Open vials policy not applicable for Hep.-B, PCV, DPT, TD, and Pentavalent
    b. Open vials policy applicable to BCG, JE, MR and Rota virus
    c. Date and time must be written after opening the vials

Don't Know

Additional Questions (during Endline survey)

22. Do you have the immunization wheel?

a. Yes

b. No

23. Can you explain what the immunization wheel is used for?
    a. To know/inform the vaccination date from the child's date of birth
    b. To provide information related to immunization
    c. To convey messages from ASHA/AWW/ANM/Gonvernment
    d. To make a due-list
    e. Other use
    f. Don't know

24. How do you use the immunization wheel?

   a. To know/inform the vaccination date from the child's date of birth
   b. To explain information to women/mothers
   c. To give to women/mothers for use at home
   d. To give the four key messages
   e. For their own reference
   f. Only to hang in the premises
   g. To make a due list
   h. Do not use it

25. Can you show the immunization wheel?

   a. Yes
   b. No

26. Where was the immunization wheel kept?

   a. It was hanging on the wall
   b. It was in the bag/purse
   c. It was in register
   d. It was kept on the table/desk
   e. It was kept in a cupboard or rack
   f. It was kept in a place away from reach
   g. Other

27. What did you like about the immunization wheel?

   a. Colour or appearance-related feature
   b. Design-related feature
   c. Immunization information-related feature
   d. Usability-related feature
   e. Other

28. What did you not like about the immunization wheel?

   a. Colour or appearance-related feature
   b. Design-related feature
   c. Immunization information-related feature
   d. Usability-related feature
   e. Other

29. Have you seen mothers (or fathers) in your village using the immunization wheel?

   a. Yes
   b. No

# ASHA and AWW RI Wheel Survey

1. Name of District:-
   a. Harda
   b. Hosangabad

2. Block Name
   a. Kharikiya
   b. Hardah/Handiya
   c. Shivani-Malwa
   d. Keshla

3. Village Name………………….
4. ASHA/ AWW Name……………..
5. Name of Surveyor………………….
6. Consent given
   a. Yes
   b. No

7. Are you an ASHA or Anganwadi worker
   a. ASHA
   b. AWW

8. Do you know the children are vaccinated?
   a. Yes
   b. No
   c. May be
   d. Cannot tell

9. Can you tell me what is vaccination? (Vaccination is the process by which a person is made immune or resistant against an infection, usually by administering a vaccine, which subsequently induces the person's own immune system to protect them against the disease.)
   a. Yes
   b. No
   c. May be

10. Can you tell me when children get their first vaccination?
    a. Correct (after birth)
    b. Wrong

c. Don’t know
d. Cannot tell

11. Can you tell me till what age children are vaccinated?
    a. Correct (5 or 16 years)
    b. Wrong
    c. Don’t know
    d. Cannot tell

12. Can you tell me which vaccines the child gets? (Mark all the correct answers and if one is told, ask someone else.)

    a. Polio
    b. BCG
    c. Hep. B
    d. Rota
    e. FIPV
    f. PCV
    g. Pentavalent
    h. MR
    i. JE
    j. DPT
    k. TD

13. Can you tell me when children are given vaccinations in the first year?(Mark all the time stated)
    a. 1.5 month
    b. 2.5 month
    c. 3.5 month
    d. 9 month
    e. Don’t know

14. Can you tell me when the child is given vaccinations after the first year? (Mark all the time stated)
    a. 16-24 Months
    b. 5 years
    c. 10 years
    d. 16 years
    e. Don’t know

15. Can you tell me how many times a mother has to come to get her child vaccinated in 5 years(5 saal 7 baar)
    a. Correct ( 6 ya 7)
    b. Wrong

c. Don’t know

16. How many times should the vaccination be done in 5 years? Is any message given to the mother regarding this? Can you tell me what that message is?
    Correct (5 saal 7 baar)
    a. Wrong
    b. Don’t know

17. Can you tell me if any vaccine is given to pregnant women?
    a. Yes
    b. No
    c. May be.
    d. Don’t know

18. Are there any myths or rumours about vaccination among people in your area?
    a. Yes
    b. No
    c. Maybe.
    d. Don’t know

19. I am going to read some myths and beliefs that people have recognized in another area. Can you tell me if you have heard them in your area? Have you heard that? Mark the correct answer
    a. Vaccinations have harmful side effects
    b. Children can not be vaccinated against whooping cough
    c. Traditional medicine has an alternative to vaccines
    d. Vaccination is not free
    e. Any others

20. Do you have any list or record of people who got vaccinated in your village?
    a. Yes
    b. No
    c. May be.
    d. Don’t know

21. I answered yes to the previous question so please ask if I can see the list. If yes is not answered in the previous question, then prompt for the name of the record and ask to show the vaccination list, if updated (due list, register, etc.)
    a. Updated
    b. Not updated
    c. Not shown

Additional Questions (during Endline survey)

30. Do you have the immunization wheel?
    a. Yes
    b. No

31. Can you explain what the immunization wheel is used for?
    1. To know/inform the vaccination date from the child's date of birth
    2. To provide information related to immunization
    3. To convey messages from ASHA/AWW/ANM/Gonvernment
    4. To make a due-list
    5. Other use
    6. Don't know

32. How do you use the immunization wheel?
    a. To know/inform the vaccination date from the child's date of birth
    b. To explain information to women/mothers
    c. To give to women/mothers for use at home
    d. To give the four key messages
    e. For their own reference
    f. Only to hang in the premises
    g. To make a due list
    h. Do not use it

33. Can you show the immunization wheel?
    a. Yes
    b. No

34. Where was the immunization wheel kept?
    a. It was hanging on the wall
    b. It was in the bag/purse
    c. It was in register
    d. It was kept on the table/desk
    e. It was kept in a cupboard or rack
    f. It was kept in a place away from reach
    g. Other

35. What did you like about the immunization wheel?
    a. Colour or appearance-related feature
    b. Design-related feature
    c. Immunization information-related feature
    d. Usability-related feature
    e. Other

36. What did you not like about the immunization wheel?
    a. Colour or appearance-related feature
    b. Design-related feature
    c. Immunization information-related feature
    d. Usability-related feature
    e. Other

37. Have you seen mothers (or fathers) in your village using the immunization wheel?
    a. Yes
    b. No

# Caregiver RI Wheel Survey

1. Name of District:-
    a. Harda
    b. Hosangabad

2. Block Name
    a. Kharikiya
    b. Hardah/Handiya
    c. Shivani-Malwa
    d. Keshla

3. Village Name

4. Tola Name

5. ASHA, AWW, ANM Name

6. What is your name?

7. What is your age?

8. How many children do you have?

9. Is any of your children younger than 9 months? Yes or No
10. How old is your child?
    a. Month
    b. 1-3 Month
    c. 3-6 Month
    d. 6-9 Month

11. Where was the child born?
    a. Govt. Hospital
    b. Pvt. Hospital
    c. Home
    d. Others

12. So have you got all your children vaccinated?
    a. Yes
    b. No

13. Where to get your children vaccinated
    a. AWC
    b. Hospital
    c. Health Sub center
    d. Pvt. Hospital
    e. Not vaccinated
    f. At Home

14. Can you tell me the date of your child's last vaccination?
    a. Tell
    b. Could not tell

15. Where did you get your vaccination information?
    a. Asha told 1 day before the session
    b. ANM told on the day of the session
    c. I found from MCP card
    d. Got from Radio, TV or posters
    e. I remembered the date
    f. I asked to ASHA
    g. I did not get the information
    h. found out from someone else

16. When is vaccination done in your village?
    a. Right (Tuesday or Friday)
    b. Don’t know

17. Can you tell me what is vaccination? (Vaccination is the process by which a person is made immune or resistant against an infection, usually by administering a vaccine, which subsequently induces the person's own immune system to protect them against the disease.)
    a. Yes
    b. No
    c. May be

18. What are the benefits of vaccination
    a. Prevents life-threatening diseases
    b. Protects children
    c. Makes children healthy and fit.
    d. Don’t know

19. Can you tell me when children get their first vaccination?
    a. Correct (after birth)
    b. Wrong
    c. Don't know
    d. cannot tell

20. Can you tell me till what age children are vaccinated?
    a. Correct (5 or 16 years)
    b. Wrong
    c. Don't know
    d. can not tell

21. Can you tell me which vaccines the child gets? (Mark all the correct answers and if one is told, ask someone else.)
    a. Polio
    b. BCG
    c. Hep. B
    d. Rota
    e. FIPV
    f. PCV
    g. Pentavalent
    h. MR
    i. JE
    j. DPT
    k. TD

22. Does the child get any vaccination immediately after birth?
    a. Yes
    b. No
    c. May be
    d. cannot tell

23. How many vaccines are administered immediately after the Birth?
    a. Correct (3)
    b. Less Number( 1 or 2)
    c. Wrong
    d. cannot tell
    e. no vaccine

24. Can you tell me when children are given vaccinations in the first year?(Mark all the time stated)
    a. 1.5 month
    b. 2.5 month
    c. 3.5 month
    d. 9 month
    e. Don't know

25. Can you tell me when the child is given vaccinations after the first year? (Mark all the time stated)
    a. 16-24 Months
    b. 5 years
    c. 10 years
    d. 16 years
    e. Don't know

26. Can you tell me how many times a mother has to come to get her child vaccinated in 5 years(5 saal 7 baar)
    a. Correct ( 6 ya 7)
    b. Wrong
    c. Don't know

27. Can you tell me if any vaccine is given to pregnant women?
    a. Yes
    b. No
    c. May be.
    d. Don't know

28. Do you know which vaccine is given to a pregnant woman? (correct answer TD/TT)
    a. Yes
    b. No
    c. Don't know

29. Do you think vaccination is safe?
    a. Yes
    b. No

30. Is it normal to have fever or swelling after vaccination?
    a. Yes
    b. No
    c. Maybe.
    d. Don't know
    e. cannot tell

31. Can the child be vaccinated when he has a cold or cough?
    a. Yes
    b. No
    c. Maybe.
    d. Don't know
    e. cannot tell

32. Are there any myths or rumours about vaccination among people in your area?

a. Yes
b. No
c. Maybe.
d. Cannot tell

33. I am going to read some myths and beliefs, that people have recognized in other area, Can you tell me if you have heard them in the your area. Have you heard that? Mark the correct answer
    a. Vaccinations have harmful side effects
    b. Children can not be vaccinated against whooping cough
    c. Traditional medicine has an alternative to vaccines
    d. Vaccination is not free
    e. Any others

34. Do you know Asha's name?
    a. Correct
    b. Wrong
    c. Don't know

35. Do you know the names of Anganwadi workers
    a. Correct
    b. Wrong
    c. Don't know

36. Do you know the name of the ANM?

    a. Correct
    b. Wrong
    c. Don't know

37. Do you think there is a need for a reminder to get your vaccination done?
    a. Yes
    b. No
    c. Maybe.

38. Can you show me your MCP card
    a. Yes
    b. No card
    c. Don't show
    d. can't find it
    e. ASHA/ANM has a card

39. If MCP card has been shown, then match the date of the last vaccination of the child with the card
    a. It is correct,

b. it is not correct,
c. there is no complete information in card

40. Note the child's date of birth from the MCP card

41. Antigen mark of 1.5 month after seeing MCP card
    a. 5 vaccine
    b. < 5 vaccine
    c. 0 vaccine

42. Note the date of vaccinations of 1.5 month by seeing at MCP card

43. antigen mark of 2.5 month after seeing MCP card

    a. 3 vaccine
    b. < 3 vaccine
    c. 0 vaccine

44. Note the date of vaccinations of 2.5 month by seeing at MCP card

45. antigen mark of 3.5 month after seeing MCP card

    a. 5 vaccine
    b. < 5 vaccine
    c. 0 vaccine

46. Note the date of vaccinations of 3.5 month by seeing at MCP card

47. Antigen mark of 9 month after seeing MCP card
    a. Two vaccines in addition to JE
    b. 1 vaccine
    c. 0 vaccine

48. Note the date of vaccinations of 9 month by seeing at MCP card

Additional Questions (during Endline survey)

49. Do you have the immunization wheel?

    a. Yes

    b. No

50. Where did you get the immunization wheel from?

a. ASHA/AWW
b. ANM
c. At the time of delivery
d. From hospital
e. From elsewhere

51. Can you explain what the immunization wheel is used for?

a. Used as a reminder for the vaccination date
b. To provide information related to immunization
c. To convey messages from ASHA/AWW/ANM/Government
d. For other purposes
e. Don’t know

52. Can you show the immunization wheel?

a. Yes

b. No

44. Where was the immunization wheel kept in the house?

a. It was hanging on the wall

b. It was hanging on the wall and was not visible

c. It was kept in a visible place

d. It was not kept in a visible place

45. What did you like about the immunization wheel?

a. Colour or appearance-related feature

b. Design-related feature

c. Immunization information-related feature

d. Usability-related feature

e. Other

46. What did you not like about the immunization wheel?

a. Colour or appearance-related feature

b. Design-related feature

c. Immunization information-related feature

d. Usability-related feature

e. Other

47. Did you use the immunization wheel to check your child's vaccination date?

a. Yes

b. No

**CLIENT EXIT CONSENT FORM**

Informed Consent

Introduction: I am part of a team conducting a study for the Clinton Health Access Initiative (CHAI). The purpose of the study is to understand the state of immunization services in the state.

Purpose of the Study: Through this study, we want to understand the quality of services provided to you.

What you are being asked to do: We would like to get your consent so that we can ask you some questions about your experience with immunization services.

Risk/Discomfort: We estimate that you may spend about 30 minutes with us. If there are any questions you do not wish to have answered you can refuse to answer without consequence. Your name or identity will not be associated with any information collected. It is possible that someone may see or hear us, however we will take all necessary precautions.

Benefits of participation: This study will result in interventions to improve immunisation services. However, there is no immediate or direct benefit to you for taking part.

Withdrawal: People who take part in this research do so voluntarily. At any time, you may end an interview or request that interview data be deleted from the study. There will be no consequences if you do not wish to be in the study. Confidentiality: Every effort will be made to ensure that your participation in this study, and all records about your participation, remain confidential. All data will be stored in secure locations and will only be available for the study team to access.

Contact Number: If you have any questions now I will answer them, and if you have questions later please contact me.

For questions about the study, please contact:

Nintin Kothari

Clinton Health Access Initiative

Phone - 9827005853 Email: nkothari@clintonhealthaccess.org

Q: Do you have any further questions?

Please take a moment to read the consent form once and you can reach out to me in person to discuss anything you may have about participating in this study and I will be happy to answer your concerns.

Consent: If I have answered all of your questions, do you agree to take part in this study?

Yes ------------------------ 1 No -------------------- 2

(Interviewer must give written consent, write down respondents' responses and then sign below)

Participant would like to receive a copy of the consent form

Yes ------------------------ 1 No -------------------- 2

Participant Signature: ________________________

Interviewer Signature: ________________________

Date: Category: Cannot Sign/Refuse to Sign:
